# ABCA8-positive lipid-metabolic CAFs mediate immunotherapy resistance in TNBC

**DOI:** 10.3389/fonc.2025.1729275

**Published:** 2026-01-27

**Authors:** Weidong Qin, Danxi Li, Jiawei Zhang, Shuning Wang, Lan Hou, Cun Zhang, Donghui Wang, Juliang Zhang

**Affiliations:** 1 Department of Epidemiology, Ministry of Education Key Lab of Hazard Assessment and Control in Special Operational Environment, School of Public Health, The Fourth Military Medical University, Xi’an, China; 2 Department of Thyroid, Breast, and Vascular Surgery, Xijing Hospital, The Fourth Military Medical University, Xi’an, China; 3 JiangXi Medical College of Nanchang University, NanChang, China; 4 State Key Laboratory of Holistic Integrative Management of Gastrointestinal Cancers, Biotechnology Center, School of Pharmacy, The Fourth Military Medical University, Xi’an, China

**Keywords:** ABCA8, cancer-associated fibroblasts (CAFs), immunotherapy, lipid, TNBC

## Abstract

**Background:**

Triple-negative breast cancer (TNBC) is an aggressive subtype characterized by the absence of estrogen receptor, progesterone receptor, and HER2 expression, which limits the availability of targeted therapies and results in poor prognosis. Immune checkpoint blockade (ICB) therapies have emerged as promising treatments by enhancing anti-tumor immunity; however, a substantial proportion of patients with TNBC exhibit primary or acquired resistance. This resistance is largely influenced by the tumor microenvironment (TME). This study uses integrated single-cell and spatial transcriptomics to elucidate key cellular mechanisms of resistance, with particular emphasis on lipid-mediated stromal-immune interactions within the TNBC TME.

**Methods:**

This investigation encompassed analysis of single-cell RNA sequencing (scRNA-seq) data from three TNBC datasets and spatial transcriptomic data from 43 TNBC samples. Spatial niches and cell-cell interactions were identified using the Multimodal Intersection Analysis (MIA) algorithm. Experimentally, adipose-derived mesenchymal stem cells (AD-SCs) were co-cultured with MDA-MB-231 TNBC cells to generate lipid-processing CAFs (lpCAFs) and subsequently co-cultured with THP-1 macrophages. Lipid metabolism and M2 polarization of macrophages were assessed using BODIPY staining, Oil Red O, qPCR, flow cytometry and Western blotting techniques.

**Results:**

ABCA8^+^ lpCAFs and APOE^+^ lipid-associated macrophages (LAMs) exhibited significant enrichment in ICB-resistant TNBC, with co-localization at the immune-stromal junction. lpCAFs facilitated M2 macrophage polarization through lipid metabolism reprogramming, establishing an immunosuppressive TME. High ABCA8 expression demonstrated correlation with enhanced M2 macrophage infiltration, decreased cytotoxic immune cells, and poorer prognosis. Experimental validation demonstrated that lpCAFs increased expression of lipid metabolism and M2 polarization marker in macrophages, substantiating their immunosuppressive function.

**Conclusion:**

ABCA8^+^ lpCAFs and APOE^+^ LAMs contribute to ICB resistance in TNBC through the establishment of an immunosuppressive TME via lipid metabolism reprogramming. Therapeutic intervention targeting the ABCA8-lipid axis presents a promising strategy to enhance ICB efficacy, potentially advancing TNBC treatment outcomes and improving patient survival.

## Introduction

Triple-negative breast cancer (TNBC) accounts for approximately 15%–20% of all breast cancer cases worldwide and is associated with disproportionately high mortality rates because of its aggressive clinical behavior and limited effective therapeutic options (1). This serious clinical challenge emphasizes the urgent need for enhanced understanding of the biological mechanisms underlying TNBC progression and treatment resistance, which could facilitate the development of more effective interventions and improved patient outcomes.

The lack of targeting interventions renders TNBC unresponsive to targeted hormonal or HER2-directed therapies. Treatment primarily depends on conventional chemotherapy and, more recently, immune checkpoint blockade (ICB) therapies, particularly anti-PD-1/PD-L1 agents, which aim to enhance anti-tumor immune responses (2). The tumor microenvironment (TME) plays a crucial role in TNBC biology, comprising a diverse array of cellular components such as immune cells, stromal cells, and extracellular matrix that collectively influence tumor growth, invasion, and immune evasion (3). Within the TME, lipid metabolic reprogramming has emerged as a key factor regulating immune cell function. Lipids, serving as energy sources, membrane components, and signaling molecules, exert a dual influence on both tumor cells and immune cells. On one hand, tumor cells upregulate lipogenic enzymes, such as fatty acid synthase (FASN), to fuel their proliferation. On the other hand, aberrant lipid metabolism in immune cells can lead to their dysfunction. For instance, the cholesterol metabolism pathway is crucial for the stability and suppressive function of Tregs (4). Concurrently, lipid peroxidation in CD8+ T cells may induce cellular exhaustion (5). Furthermore, fatty acid oxidation (FAO) and cholesterol efflux in TAMs drive their polarization towards an immunosuppressive M2 phenotype (6). These findings underscore the central role of lipid metabolism in shaping an immunosuppressive TME.

Cancer-associated fibroblasts (CAFs) and tumor-associated macrophages (TAMs) are particularly critical in the TME. CAFs promote fibrosis and extracellular matrix remodeling, whereas TAMs often adopt immunosuppressive phenotypes that compromise effective anti-tumor immune surveillance (7, 8). Recent developments in single-cell RNA sequencing (scRNA-seq) and spatial transcriptomics have revealed the marked heterogeneity of CAFs and TAMs, identifying subsets involved in immune escape and tumor progression (9, 10).The formation of an immunosuppressive microenvironment depends on specific spatial structures. Further exploration of neighboring effects and intercellular communication will contribute to unraveling the challenges of immune evasion (11).

This study integrates scRNA-seq and spatial transcriptomic data from large-scale TNBC cohorts to comprehensively characterize the TME composition, with a focus on identifying CAFs and TAMs subsets associated with immunotherapy resistance. Our study demonstrates for the first time in TNBC that lpCAFs, via lipid efflux mediated by their marker ABCA8(ATP Binding Cassette Subfamily A Member 8)—an ATP-binding cassette (ABC) family membrane transporter involved in cholesterol and phospholipid efflux—induce metabolic reprogramming and promote the polarization of macrophages toward an immunosuppressive phenotype. The results of our study include the following aspects: (1) delineating spatial niches, (2) analyzing subset enrichment in resistant cases, and (3) validating key functional mechanisms using *in vitro* models and patient-derived tissue samples. Through these findings, we have identified novel strategies that can improve the clinical outcomes of patients with TNBC.

## Methods

### Single-cell transcriptome analysis and differential expression analysis

scRNA-seq data were analyzed using the Scanpy pipeline (v1.9.8). Raw counts underwent log-normalization and were subsequently used to identify 3,000 highly variable genes (12). For dimensionality reduction and batch correction, the raw counts of these highly variable genes were processed using scvi-tools (v0.17.1), generating 50 latent dimensions. These latent dimensions were then used to construct a KNN graph for uniform manifold approximation and projection (UMAP) visualization and Leiden clustering.

A comprehensive quality control workflow was applied to the samples. Scrublet was used to identify and remove potential doublets from each sample, and DecontX was employed to eliminate ambient RNA contamination (13, 14). Cells with fewer than 1,000 counts or exceeding 15% mitochondrial gene content were excluded from the analysis. To further minimize batch effects caused by low-quality samples, we excluded samples containing fewer than 1000 cells. In addition, to prevent patient-specific effects from disproportionately influencing the analysis, the Harmony algorithm was applied for batch correction across all scRNA-seq samples.

### Cell type annotation

The major cell types were identified by examining the expression patterns of well-established marker genes. Cell subtypes were further defined through unsupervised clustering and analysis of marker gene differential expression, as shown in the figures. Differentially expressed genes (DEGs) in each subcluster were identified using Seurat’s “FindAllMarkers” with default parameters. These DEGs were then used to annotate cell subtypes and to perform pathway enrichment analysis.

### Pathway analysis

Differential gene analysis among the CAF subtypes was performed using the FindAllMarkers function. In addition, the FindMarkers function was applied to identify differentially expressed genes between the expanded (E) and non-expanded (NE) groups of T and NK cells. Gene Set Enrichment Analysis (GSEA) was then performed on the differentially expressed gene sets using the clusterProfiler R package (V.4.7.1.2). The NES and p-values are shown in the figures.

### Spatial transcriptomic analysis

The SCTransform method from the Seurat package was utilized to standardize gene expression counts of spatial transcriptomics spots (15). The ‘anchor’-based integration workflow from Seurat was implemented to integrate transcriptomic data across all 43 samples. For anchor identification, 3,000 integration genes were selected. Dimensionality reduction was performed using RunPCA (PC = 30) and RunUMAP (resolution = 0.3). The FindAllMarkers function was then used to identify characteristic genes for each cluster in the spatial transcriptome and for each cell type in the scRNA-seq data.

To visualize and analyze spatial relationships of specific genes and cell types, the spatial analysis tools Semla and Squidpy were employed (16, 17).

Tissue dissociation prior to scRNA-seq can lead to spatial information loss, while the spatial transcriptome cannot achieve cellular resolution. Multimodal Intersection Analysis (MIA), a hypergeometric distribution test method, was implemented to integrate spatial and scRNA-seq analysis results, thereby annotating the precise cellular composition of distinct tissue regions (18). Niches in spatial regions were defined as primary areas for specific cell types and were enriched with the characteristic genes of the corresponding cell type.

The spatial co-localization analysis was conducted using the method proposed by Liu, Q et al., with the code available at https://genome.cshlp.org/content/suppl/2022/09/19/gr.276851.122.DC1 (19). We calculated the expression values of ABCA8 (representative gene of lpCAFs), CD68 and APOE (representative genes of LAM,mean value), for each spot on the spatial transcriptome chip. Spots with expression levels in the top 10% were defined as high-expressing. The calculation of the colocalization score integrates ligand-receptor gene expression levels with spatial proximity. Specifically, based on the original coordinates of each spatial spot, the K-nearest neighbor (KNN) algorithm (K = 6 in the code) is first used to identify neighboring spots for each spot (excluding itself). Subsequently, the expression levels of ligand and receptor genes are normalized to eliminate differences in sequencing depth among different spots, yielding the intrinsic ligand/receptor expression level of each spot and the maximum receptor/ligand expression level among its neighboring spots, respectively. Finally, the core formula pmax(intrinsic ligand expression × maximum neighboring receptor expression, intrinsic receptor expression × maximum neighboring ligand expression) is applied to calculate the score, where the maximum value of the two terms is taken as the colocalization score for the spot. Meanwhile, extreme values are trimmed to avoid abnormal impacts. The resulting score reflects the potential for spatial interactions between ligands and receptors in the spot and its adjacent regions.

### High-dimensional weighted gene co-expression network analysis

hdWGCNA is an R package designed to perform WGCNA on high-dimensional transcriptomics data, including single-cell RNA-seq. The package is highly modular and can construct context-specific co-expression networks across cellular and spatial hierarchies (20). In this study, the hdWGCNA algorithm was employed to analyze co-expression networks of genes in CAFs. Module detection was performed using the dynamic tree cutting method with a recommended soft thresholding power. The M2 module is relatively highly expressed in lpCAF, and we use the top 30 genes of the M2 module as marker genes for lpCAF.

### Bulk RNA transcriptomic analysis

Kaplan-Meier plots were used to assess the effects of ABCA8 on the prognosis of patients with TNBC, parameter settings are as follows: ER status-IHC: ER-negative; PR status-IHC: PR-negative; HER2 status-array: HER2-negative; dataset: all (https://kmplot.com/analysis/) (21). In addition, TIMER3.0 (https://compbio.cn/timer3/) was used to calculate the correlation between ABCA8 expression and the infiltration levels of specific immune cells and CAFs (22).

### Extraction and identification of ADSCs

We extracted adipose tissue from discarded breast tissue (non-tumor tissue) of patients who underwent mastectomy. The adipose tissue was cut into approximately 1 mm³ microblocks and mixed with an equal volume of 0.1% or 0.2% type I collagenase solution. After sealing, the mixture was incubated in a 37°C water bath for 30 minutes, with shaking every 5 or 10 minutes until liquefied. A 70-mesh filter was used to remove residual tissue, followed by centrifugation at 300g and supernatant removal. The cells were washed with PBS, centrifuged at 300g, and the supernatant was discarded. The cells were resuspended in DMEM medium containing 10% fetal bovine serum (Gibco, USA) and inoculated into culture dishes. Cell culture was performed in a 37°C incubator with 5% CO_2_. After 48 hours, the culture medium was replaced, and the obtained cells were primary ADSCs. Cell identification was performed using flow cytometry.

### MDA-MB-231 and ADSCs co-cultivation for adipogenesis induction

ADSCs were inoculated in the lower layer of 6-well Transwell chambers (Corning, USA). MDA-MB-231 cells were then seeded in the upper layer of Transwell co-culture, while the upper layer of single culture remained unseeded. Upon reaching 90% ADSCs density, the culture medium was replaced with adipogenesis induction medium, and co-culture was maintained for 14 days (23).

### BODIPY staining

The samples were first rinsed twice with PBS and then fixed in 4% paraformaldehyde for 30 minutes at room temperature. After fixation, the samples were washed twice with PBS and incubated in a BODIPY 493/503 solution (Shanghai Maokang) at a final concentration of 5 μM for 30 minutes at 37°C in the dark. The stain was removed after incubation, and the samples were rinsed twice with PBS (24, 25). Stained samples were imaged using a Thermo Fisher EVOS M7000 fluorescence microscope. For each field of view, both the BODIPY fluorescence image and the corresponding brightfield image were captured using a 20× objective. Quantitative analysis was performed using ImageJ software (26, 27).

### Oil red O staining and quantification

Samples were first rinsed twice with PBS and then fixed in 4% paraformaldehyde for 10 minutes. After removing the fixative, the samples were washed twice with PBS. A small amount of 60% isopropanol was added to cover the cells for 15–20 seconds, after which the isopropanol was removed. The cells were then incubated with Oil Red O working solution (ServiceBio) at room temperature in the dark for 30 minutes. The staining solution was removed, and 60% isopropanol was added for 3–5 seconds to achieve rapid differentiation. Samples were washed three times with distilled water (5 minutes each time) and rinsed twice with PBS. Stained samples were observed under a Thermo Fisher EVOS M7000 microscope, and brightfield images were captured using a 20× objective. Quantitative analysis was performed using ImageJ software. The Oil Red O-positive area was measured and expressed as a percentage of the total cellular area identified in the same field (28, 29).

### Western blotting

Cells were lysed using RIPA lysis buffer (Thermo Fisher). The supernatant was frozen at 4°C for 30 minutes, followed by centrifugation at 4°Cg and 12,000 r/min for 30 minutes to collect the pellet. The total protein concentration was determined using a BCA kit (Beyotime). A 40μg protein sample was loaded onto an SDS-PAGE gel. The separated proteins were transferred to a polyvinylidene fluoride (PVDF) membrane (Thermo Fisher), which was blocked with 5% skim milk for 1 hour. The membrane was incubated overnight with primary antibodies. Following a 1-hour secondary antibody incubation, protein detection was performed using the enhanced chemiluminescence (ECL) kit (Thermo Fisher). The membrane underwent three TBST buffer washes between each step. Quantitative analysis was conducted using ImageJ software. Detailed antibody specifications are presented in **Supplementary Table S1**.

### Real-time quantitative PCR

RT-qPCR was performed to analyze the gene expression levels of ABCA8 and APOE. Total mRNA was extracted from cells in each group using Trizol reagent (Invitrogen, USA), followed by centrifugation and DEPC treatment for concentration determination. The mRNA was reverse transcribed into cDNA using the PrimeScript FAST RT Kit (TAKARA, Japan). RT-qPCR analysis was conducted on a C1000 RT-PCR system (BIO-RAD, USA) using the synthesized cDNA and TB Green Premix Ex Taq II FAST qPCR (TAKARA, Japan) according to the manufacturer’s protocols. Glyceraldehyde-3-phosphate dehydrogenase (GAPDH) was used as the housekeeping gene. Gene expression levels were quantified using the ΔΔCt method, normalizing target gene expression relative to the housekeeping gene. The primer sequences are listed in **Supplementary Table S2**.

### siRNA transfection

lpCAF cells in the logarithmic growth phase were trypsinized, resuspended, and subjected to cell counting. The cell suspension was then seeded at a density of 5 × 10^5 cells per well in 6-well plates and cultured in serum-containing medium until reaching approximately 70% confluence to initiate siRNA transfection. For transfection, two tubes (A and B) were prepared, each containing 250 μL of Opti-MEM medium (Gibco, 31985070). ABCA8-siRNA (or negative control siRNA, NC-siRNA, obtained from Tsingke Biotechnology Co., Ltd) was added to tube A to achieve a final concentration of 20 nM, while 5 μL of Lipofectamine 3000 (Invitrogen, L3000001) reagent was added to tube B. After gentle mixing, both tubes were incubated at room temperature for 5 minutes, followed by combining the solutions from tubes A and B. The mixture was further incubated at room temperature for 15 minutes to form siRNA-lipid complexes. These complexes were then added dropwise to the corresponding wells of the cell culture plate. Following 6–8 hours of transfection, the old medium was aspirated and replaced with 2 mL of fresh complete medium for continued culture.

### Flow cytometry

After co-culture, macrophages were collected and pelleted by centrifugation, followed by one wash with phosphate-buffered saline (PBS). For antibody staining, cells were resuspended at a concentration of 1×10^6^ cells per 5 μL of antibody. The anti-human CD163 antibody (PE-65561), the anti-human APOE antibody (66830-1) and the isotype control antibody (65124-1-Ig) was obtained from Proteintech. Incubation was carried out at room temperature for 15 minutes in the dark (after the APOE antibody is depleted, it needs to be labeled with a fluorescent anti-mouse secondary antibody). Subsequently, cells were washed with 1 mL of PBS and centrifuged to obtain the cell pellet. Finally, the stained cells were resuspended in 200 μL of PBS and analyzed using a flow cytometer.

### LpCAFs and iCAFs co-culture with macrophages

THP-1 Induction to M0: THP-1 cells were incubated with 150 nM Phorbol 12-myristate 13-acetate (PMA, MCE) for 24–48 hours, followed by cultivation in RPMI 1640 medium for an additional 24 hours to achieve stabilization and maturation into M0 macrophages. M0 cells were placed in the lower chamber of a 6-well Transwell chamber, while lpCAFs or iCAFs were seeded in the upper chamber.

### Immunofluorescence staining

Samples were first rinsed twice with PBS, followed by fixation in 4% polyformaldehyde for 30 minutes. Cells or tissues were treated with 0.1% Triton X-100 for 10 minutes, followed by incubation with 3% bovine serum albumin (BSA, Thermo Fisher) at room temperature for 30 minutes. Primary antibody incubation was performed overnight at 4°C. After three PBS washes, the samples were incubated with a secondary antibody at room temperature for 2 hours, followed by DAPI staining. The staining results were observed under microscopic examination(Thermo Fisher EVOS M7000). Quantitative analysis was performed using ImageJ software.

### Statistical analysis

All statistical analyses were performed using SPSS 21.0 (IBM Corp., NY, USA) and GraphPad Prism 7 (v7.02; GraphPad Software, CA, USA). Quantitative data are expressed as mean ± SD from three independent biological replicates. Intergroup comparisons were conducted using two-tailed Student’s t-tests. Statistical significance was established at p < 0.05.

## Results

### scRNA-seq atlas and spatial niche analysis of TNBC

**Figure 1:**
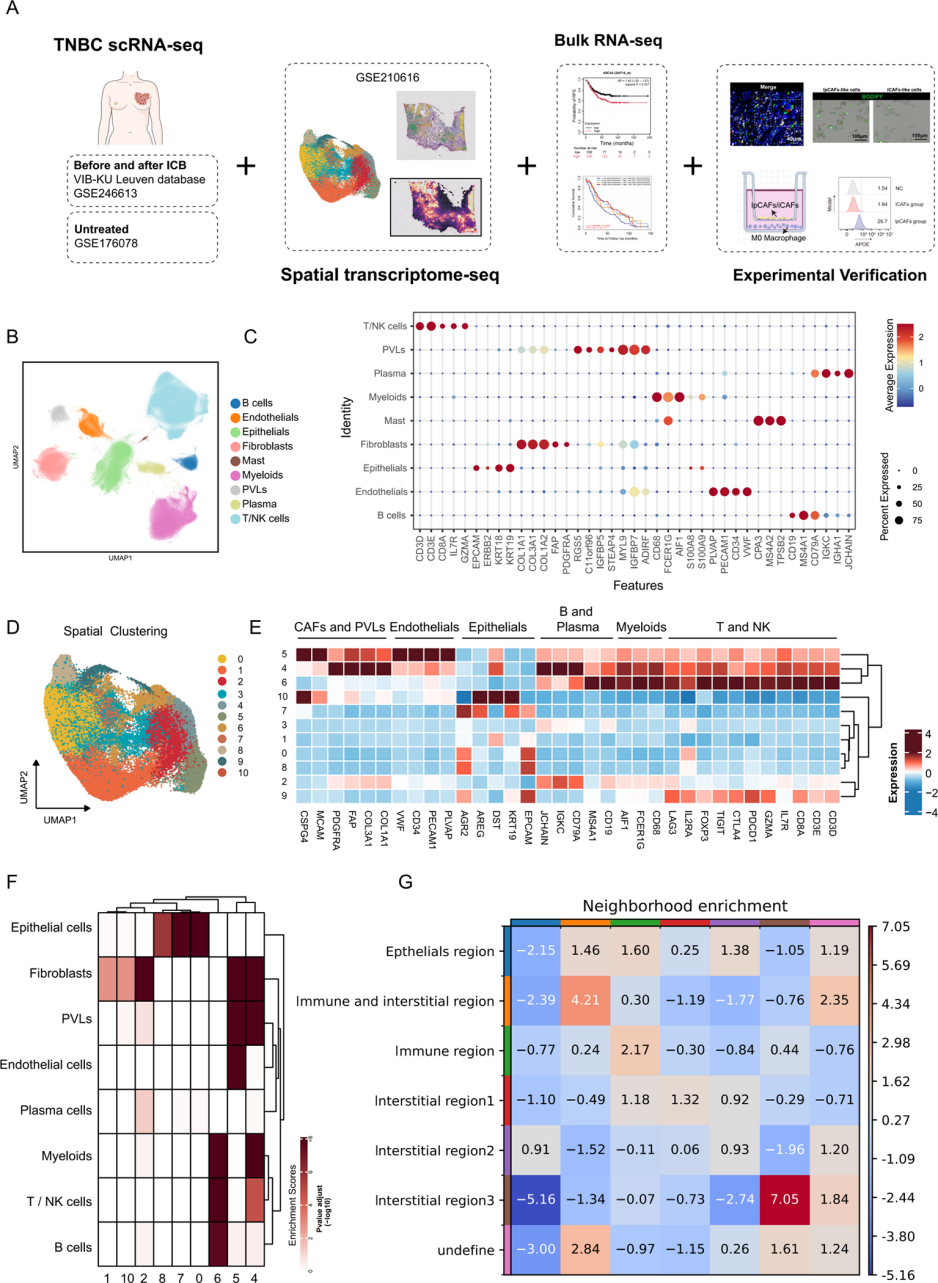
Characteristics of single-cell and spatial transcriptomics in TNBC. **(A)** Workflow integrating public databases with experimental validation. **(B)** UMAP visualization of dimensionality-reduced and unsupervised clustered cellular populations within the TNBC tumor microenvironment; distinct colors represent cell types. **(C)** Bubble plot depicting feature gene distribution across clusters. Bubble size corresponds to the percentage of cells expressing marker genes within each cluster, while the color gradient reflects normalized mean expression levels. **(D)** UMAP plot showing the results of dimensionality-reduction-based clustering of spatial transcriptomics data, with each color representing a different cluster. **(E)** Heatmap illustrating the distribution of characteristic marker genes for each cell type across different clusters. **(F)** MIA results showing the distribution of various cell types within different clusters. **(G)** Heatmap showing the enrichment scores for the spatial proximity of regions; higher scores indicate closer spatial adjacency.

To systematically analyze the composition of the TME in TNBC, this study integrated TNBC spatial transcriptomic data and scRNA-seq data to characterize the features of its tumor niche. Through scRNA-seq data analysis, specific subsets of TAMs and CAFs associated with immune therapy resistance in TNBC were identified. To investigate the mechanism by which ABCA8^+^ lpCAF potentially induces M2 polarization of macrophages through lipid metabolic reprogramming and mediates immune suppression, this study used bulk RNA-seq data from public databases, cell functional experiments, and postoperative tissue immunofluorescence staining results from patients with TNBC exhibiting immune therapy resistance (**Figure 1A**).

This study integrated three large-scale TNBC scRNA-seq datasets (30–32). After rigorous screening and quality control, 613,888 cells were included for analysis. Based on differential expression patterns of specific marker genes for major cell types, these cells were categorized into nine major subsets: epithelial cells, CAFs, endothelial cells, perivascular-like cells (PVLs), myeloid cells, T and NK cells, B cells, plasma cells, and mast cells (**Figure 1B**). The characteristic genes of each subset are illustrated through a bubble chart (**Figure 1C**). To analyze the spatial niche characteristics of TNBC, this study integrated 43 TNBC spatial transcriptomic samples from Bassiouni, R., et al.’s research (33). Dimensionality reduction and clustering identified 11 spatial regions (clusters) (**Figure 1D**). The differential expression patterns of characteristic genes of major cell types in each cluster are presented as a heat map (**Figure 1E**). Subsequently, by calculating the correlation between scRNA-seq data (cell type differential genes) and spatial regions (cluster differential genes), and applying the MIA method for significant enrichment analysis, the enrichment scores of cell types in each spatial region were determined (**Figure 1F**). Based on the MIA enrichment scores and expression distribution of characteristic genes in each spatial region, these 11 spatial regions were defined as follows: Epithelials region: highly enriched with epithelial cells (c0, c7, c8, c9); Immune region: mainly enriched with immune cells (c6); Immune and interstitial region: co-enriched with fibroblasts and immune cells (c4); Interstitial region 1: highly enriched with fibroblasts, pericytes, and endothelial cells (c5); Interstitial region 2: mainly composed of fibroblasts, with relatively enriched plasma cells (c2); Interstitial region 3: mainly composed of fibroblasts, accompanied by minimal enriched epithelial cells (c1, c10). The squidpy algorithm calculated spatial proximity enrichment scores between various niches. Results indicated that the Immune region and immune and interstitial region, containing key target cells for immunotherapy (T and NK cells), were significantly adjacent to the epithelial cell region (tumor region) in spatial distribution (**Figure 1G**).

**Figure 2:**
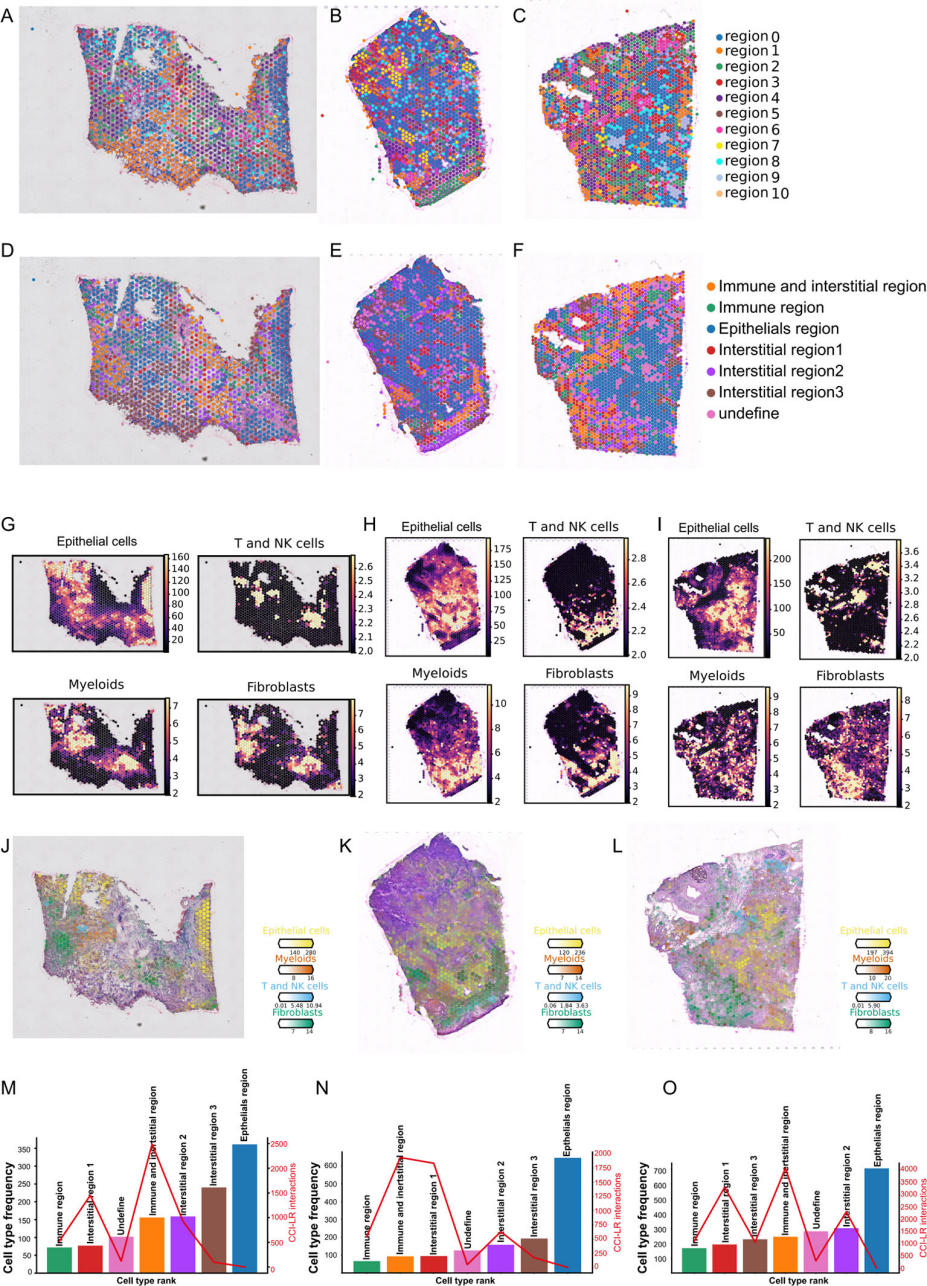
Spatial transcriptomic characteristics of representative TNBC samples. **(A-C)** H&E staining and spatial transcriptomic spot clustering results of representative TNBC spatial transcripts. **(D-F)** H&E staining and spatial transcriptomic functional region partitioning of representative TNBC spatial transcripts. **(G-I)** Deconvolutional abundance of spatial transcriptomic spots for epithelial cells, fibroblasts, myeloid cells, T and NK cells using cell2location. **(J-L)** Integrated visualization of estimated abundance (color intensity) of cell types (colors) in epithelial cells, fibroblasts, myeloid cells, T cells, and NK cells. **(M-O)** The stLearn algorithm was used to calculate the relative quantity and communication intensity of each region. The bar chart showed the relative quantity of each region (left coordinate axis), and the line chart showed the cell communication intensity of each region (right coordinate axis).

Projecting the spatial niche analysis results onto representative samples demonstrates that the Immune region and the immune and interstitial region are closely adjacent to the tumor core region (**Figures 2A-F**). Analysis using the cell2location spatial cell type deconvolution algorithm revealed that T/NK cells, myeloid cells, and fibroblasts are significantly enriched in the tumor-stroma junction region (**Figures 2G-L**). Analysis of intercellular spatial communication indicated that cell communication activity in the immune and interstitial region was significantly higher than in the tumor core region (**Figures 2M-O**). Combined with functional enrichment analysis results of differential genes in each functional region (**Supplementary Figure S1A**), these findings suggest that the immune and interstitial region serves as a key regulatory hub in the tumor microenvironment, potentially playing a crucial role in regulating tumor growth, invasion, and metastasis by promoting close interactions between immune cells and stromal cells. Detailed investigation of the cell communication mechanism in this region may provide new intervention targets for tumor treatment strategies.

### Enrichment of APOE^+^ LAM subgroup with immunotherapy resistance in TNBC​​

The spatial characterization results of TNBC revealed that T cells exist in the immune-stromal microenvironment composed of myeloid cells and fibroblasts. Previous studies have confirmed that fibroblasts and myeloid cells play a crucial role in the development of the tumor immunosuppressive microenvironment. Therefore, this study utilized two single-cell datasets containing prognostic information of neoadjuvant immunotherapy to conduct dimensionality reduction and clustering analysis on myeloid cells and fibroblasts to investigate their mechanism of action in TNBC immunotherapy.

**Figure 3:**
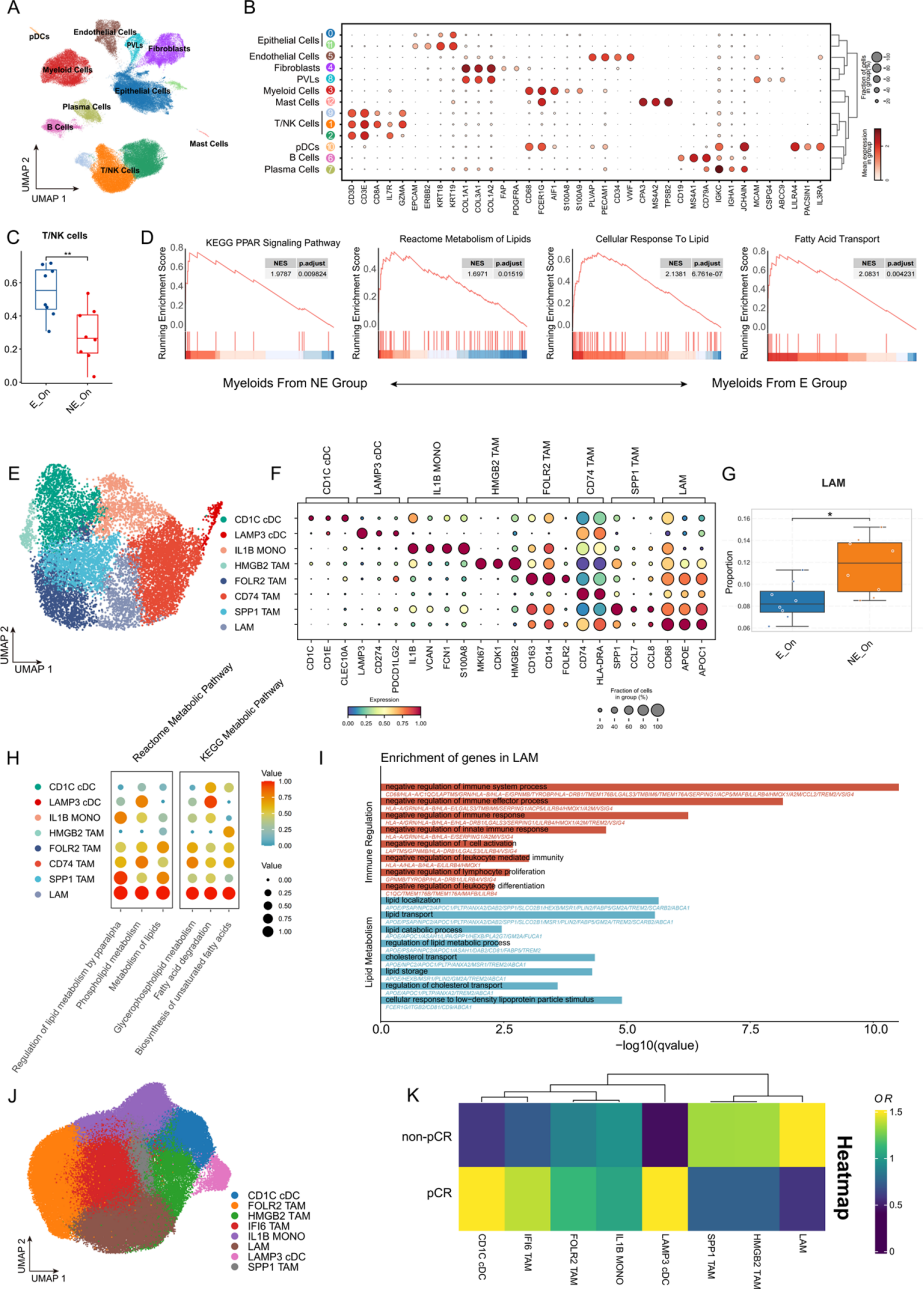
Relative enrichment of macrophages with lipid metabolism characteristics was associated with poor prognosis of immunotherapy in TNBC. **(A)** UMAP visualization of dimensionality-reduced and unsupervised clustered cellular populations within the TNBC tumor microenvironment using the datasets from the VIB - KU Leuven Cancer Biology Center. Different colors represent cell types. **(B)** Bubble plot showing feature gene distribution across clusters. Bubble size represents the percentage of cells expressing marker genes within each cluster, and the color gradient indicates normalized mean expression levels. **(C)** Box plot showing the proportion of infiltrating T and NK cells in TME of the T cell amplification group **(E)** and the non-amplification group (NE) before and after treatment, the wilcox test, ** represents p <0.001. **(D)** GSEA of myeloid cells differential genes between NE and E group (NES>1 indicates responder-enriched pathways). **(E)** UMAP projection of myeloid cells from the datasets of VIB - KU Leuven Cancer Biology Center after dimensionality reduction and unsupervised clustering, with colors distinguishing cell subtypes. **(F)** Bubble plot showing the feature gene distribution across clusters. The bubble size corresponds to the percentage of cells expressing marker genes within each cluster; the color gradient reflects normalized mean expression levels. **(G)** Box plot showing the proportion of LAM in myeloid cells in the T cell amplification group **(E)** and the non-amplification group (NE) before and after treatment, the Wilcox test, * represents p <0.05. **(H)** scMetabolism analysis of lipid metabolism-related pathways in each myeloid cell subgroup. Bubble size and the color gradient reflects the normalized mean expression level. **(I)** Bar chart showing the GO enrichment results of LAM differential genes. **(J)** UMAP projection of myeloid cells from GSE246613 after dimensionality reduction and unsupervised clustering, with colors distinguishing cell subtypes. **(K)** Heatmap showing the odds ratio (OR) of myeloid cell subtype abundance between pCR and non-pCR groups in GSE246613 dataset.

This study analyzed scRNA-seq data from the VIB-KU Leuven Cancer Biology Center database (30). TNBC samples containing immune cell expansion information and exceeding 1,000 cells were selected. Quality control yielded 92,851 high-quality cells. These patients with TNBC received pembrolizumab treatment one week before surgery, and tumor specimens were collected for scRNA-seq analysis before ICB treatment and after surgery. Following quality control and dimensionality reduction, the cells were categorized into 10 main cell types (**Figure 3A**), with each cell type identified by classic scRNA-seq marker gene expression (**Figure 3B**). Patients were stratified into an expanded (E) group, characterized by clonal expansion of T cells during treatment and associated with treatment response, and a non-expanded (NE) group, lacking such expansion and associated with non-response, based on single-cell TCR sequencing (scTCR-seq) data. The proportion of T cells and NK cells in the E group after treatment was significantly higher than that in NE groups (**Figure 3C**). This finding aligns with Bassez’s scRNA-seq analysis conclusions (which did not differentiate between breast cancer subtypes).

Differential analysis between the treated myeloid cell groups revealed that myeloid cells in the NE group exhibited significant enrichment in lipid metabolism-related pathways (**Figure 3D**). Subsequent dimensionality reduction and clustering of myeloid cells identified two dendritic cell subsets (CD1C^+^ cDC and LAMP3^+^ cDC), one monocyte subset (IL1B^+^ MONO), and five macrophage subsets: HMGB2^+^ TAM, FOLR2^+^ TAM, CD74^+^ TAM, SPP1^+^ TAM, and LAM(lipid-associated macrophages), which is APOE -positive and demonstrates lipid metabolism characteristics (**Figure 3E**). The differential expression of characteristic genes in each subset is illustrated in a bubble chart (**Figure 3F**). Analysis of myeloid cell subset proportions across treatment-responsive and non-responsive groups revealed significant LAM enrichment in the non-responsive group (**Figure 3G**; **Supplementary Figure S1B**). Metabolic analysis using the scMetabolism function confirmed higher lipid metabolism characteristics in the LAM subset (**Figure 3H**). The Findallmarkers function identified characteristic genes of each subset, and GO enrichment analysis of LAM differential genes demonstrated significant enrichment in lipid metabolism and immunosuppressive pathways, indicating its potential role in TNBC immunotherapy resistance (**Figure 3I**).

These findings were validated using scRNA-seq data from GSE246613, which analyzed single-cell sequencing of a patient with TNBC puncture biopsy samples before treatment, after one pembrolizumab treatment cycle, and following pembrolizumab combined with radiotherapy. Dimensionality reduction and myeloid cell clustering produced similar subset clustering results (**Figure 3J**), with characteristic gene distribution shown in **Supplementary Figure S1C**. Odds ratio analysis revealed that at this specific time point following PD-1 monotherapy, patients who did not achieve a pathological complete response (pCR) after complete treatment exhibited a higher relative LAM enrichment compared to those who achieved pCR (**Figure 3K**). GO enrichment analysis of subset-specific differential genes confirmed the lipid metabolism and immunosuppressive characteristics of the LAM subset (**Supplementary Figure S1D**). These results corroborate previous findings, suggesting LAM subset involvement in TNBC immunotherapy resistance. Further investigation of LAM subset formation and regulatory mechanisms may provide novel approaches for addressing TNBC immunotherapy resistance.

### Enrichment of ABCA8^+^ lpCAF subgroup associates with immunotherapy resistance in TNBC​

Previous spatial characterization of TNBC identified an enhanced cellular interaction network in the immune-stromal junction containing T cells, NK cells, myeloid cells, and CAFs. Single-cell data-based cell communication analysis demonstrated significantly increased communication signal reception by myeloid cells in the NE group, suggesting that robust CAF-myeloid cell interaction may contribute to immunotherapy resistance(**Supplementary Figure S2A**).

**Figure 4:**
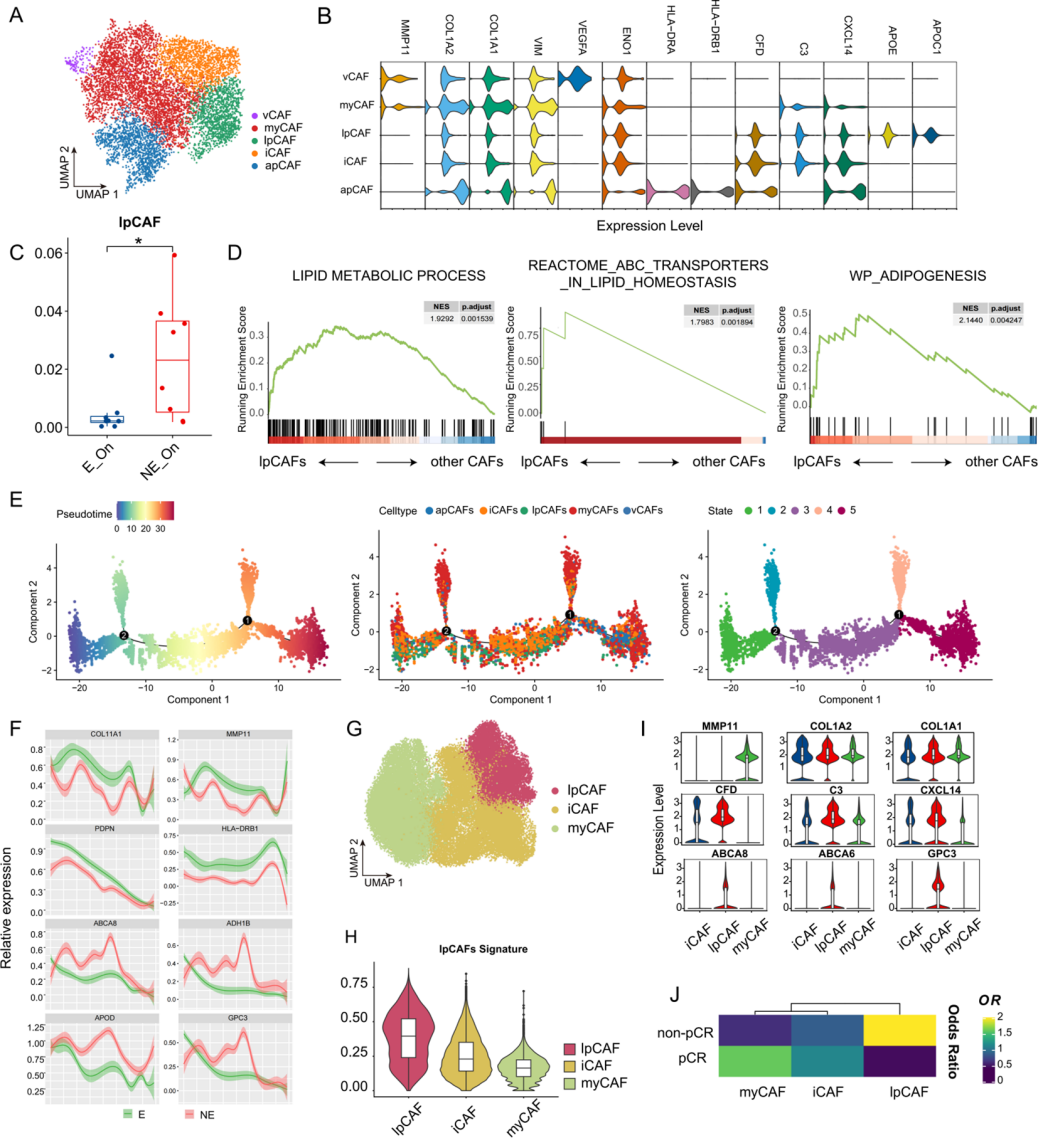
Relative enrichment of CAF cells with lipid metabolism characteristics associated with poor prognosis of immunotherapy in TNBC. **(A)** UMAP plot illustrating the classification of CAFs in TNBC. Distinct colors represent different subtypes. **(B)** Violin plot showing the expression levels of relevant marker genes in each CAF subtype. **(C)** Box plot illustrating the differential distribution of lpCAFs between the E and NE groups before and after ICB treatment. **(D)** GSEA demonstrating specified pathway differences between lpCAFs and other CAFs. Genes are sorted based on fold change between two conditions. **(E)** Developmental trajectory of CAFs, inferred by monocle 2, displays sequential coloring using pseudo time, trajectory states, and CAF subtypes. **(F)** Differential expression patterns of specified genes along a simulated time trajectory in the E and NE groups. **(G)** UMAP plot showing the dimensionality reduction clustering results of cells in the GSE246613 scRNA-seq data, with distinct colors representing different cell types. **(H)** Violin plot illustrating the distribution of the lpCAFs signature across different CAFs subclusters. **(I)** Violin plot depicting the distribution of marker genes across different CAFs subclusters. **(J)** Heatmap depictimng the OR values of different CAFs subclusters in pCR and non-pCR groups.

CAFs exhibit heterogeneity and plasticity crucial for tumor progression. Analysis of the VIB - KU Leuven Cancer Biology Center database classified CAFs into five subtypes based on gene expression profiles: antigen-presenting CAFs (apCAFs), characterized by high HLA-DRA1 and HLA-DRB1 expression; inflammation-associated CAFs (iCAFs), marked by elevated C3, CFD, and CXCL14 chemokine expression; vasculature-associated CAFs (vCAFs), featuring high angiogenesis-related VEGFA and ENO1 expression; lipid-processing CAFs (lpCAFs), distinguished by APOE and APOC1 expression; and myofibroblast-like CAFs (myCAFs), characterized by abundant extracellular matrix (ECM)-related genes including COL1A2, COL1A1, and VIM (**Figure 4A**). These subtypes were confirmed using the classic CAF markers (**Figure 4B**).

In ICB-treated patients with TNBC, the NE group showed significantly higher lpCAF proportions than the E group, suggesting a potential ICB resistance association (**Figure 4C**). Beyond expressing iCAF-related genes such as C3 and CFD, GSEA analysis revealed lpCAF enrichment in lipid metabolism and ABCA family lipid transport pathways (**Figure 4D**). Monocle 2 algorithm-based pseudotime trajectory analysis of CAFs showed similar trajectories for lpCAFs and iCAFs, predominantly in state1 and state3, distinctly different from myCAFs, concentrated in state2 and state4 (**Figure 4E**). The analysis included lipid metabolism-related marker genes (ABCA8, ADH1B, APOD, and GPC3) and other CAF subtype markers to demonstrate their dynamic changes and differential expression between the E and NE groups (**Figure 4F**).

To validate these findings, we analyzed single-cell RNA sequencing data from GSE246613. Although the original data set primarily categorized CAFs into myCAFs and iCAFs, further analysis identified a subpopulation of lpCAFs using specific marker genes (**Figure 4G**). The violin diagram demonstrates that the signatures of lpCAFs which were identified using WGCNA analysis (**Supplementary Figures S2B–D**), and the specific genes of lpCAFs can be used to distinguish lpCAFs from other CAFs subtypes (**Figures 4H, I**). Analysis of subtype distribution preference using odds ratio calculations revealed that patients who did not achieve pCR after neoadjuvant therapy showed significantly higher relative enrichment of lpCAFs following PD-1 treatment compared with patients who achieved pCR. This finding is consistent with previous observations, indicating that the lpCAF subgroup may contribute to immunotherapy resistance in TNBC (**Figure 4J**).

### Spatial proximity between ABCA8^+^ lpCAF and APOE^+^ LAM​

**Figure 5:**
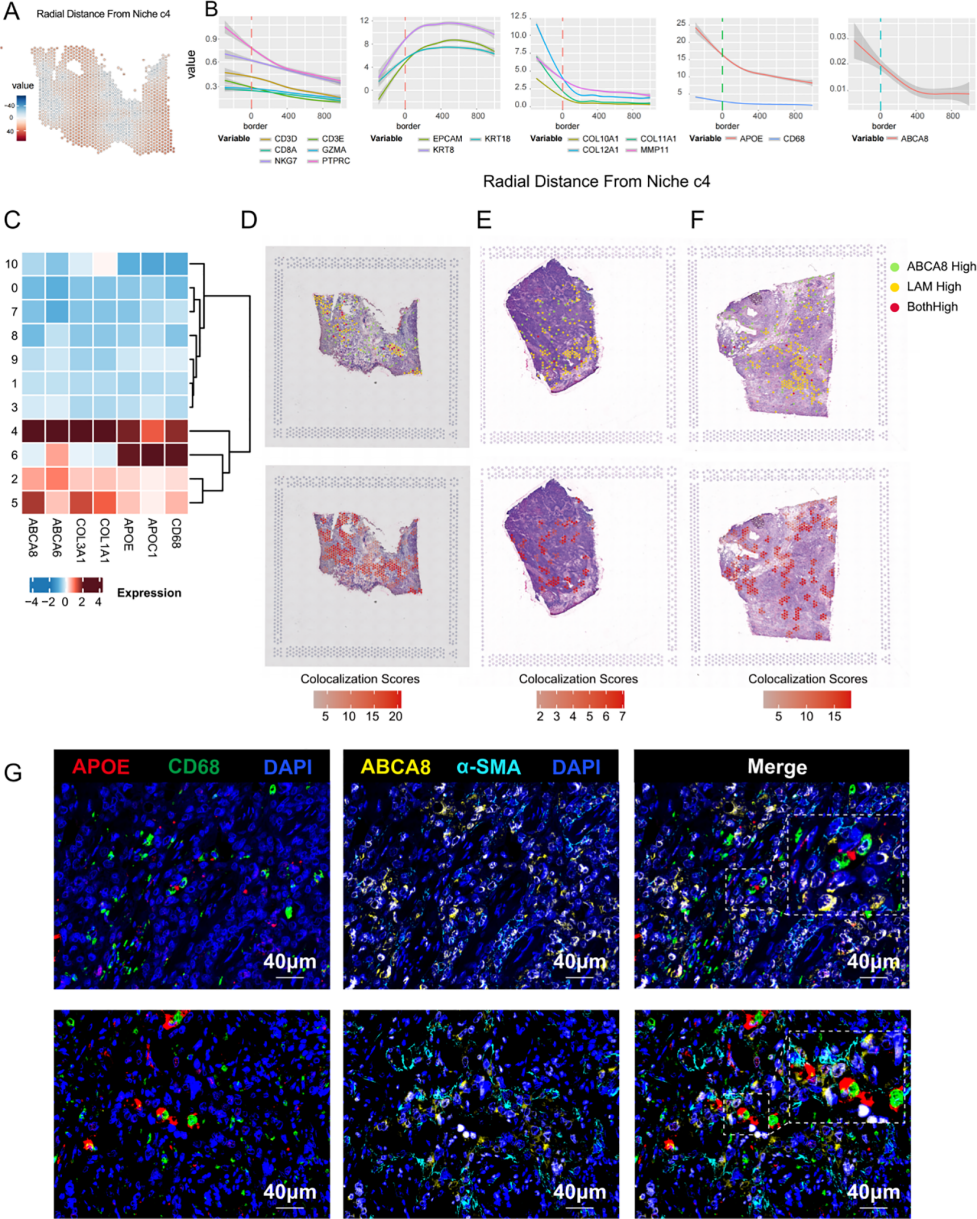
Spatial co-localization of ABCA8-positive CAF and APOE-positive LAM. **(A)** Radial relative distance between each spot and Niche c4, where positive values indicate radial distances out from the niche and negative values indicate radial distances towards the niche center. For improved visualization, the square root of the distance was used. **(B)** Relationship between expression levels of CTL marker genes, stromal marker genes, epithelial marker genes, LAM marker genes, and ABCA8 with the spatial distance relative to the stromal-immune interface niche c4. The x-axis indicates the distance relative to c4, where 0 represents the boundary region, negative values indicate the c4 region interior, and positive values indicate regions outside c4. The y-axis represents gene expression levels. **(C)** Heatmap illustrating the distribution of marker genes for ABCA8^+^ lpCAFs and APOE^+^ LAM across different regions. **(D-F)** Spatial colocalization plots showing overlap between spots with high ABCA8 expression (top 10%) and those with high expression of APOE^+^ LAM marker genes (APOE and CD68) (top 10%). **(G)** Multiplex immunofluorescence staining revealing the spatial proximity of ABCA8^+^ lpCAFs and APOE^+^ LAM. Red indicates APOE, green indicates CD68, yellow indicates ABCA8, cyan indicates α-SMA, and dark blue indicates DAPI.

Previous single-cell data analyses identified relative enrichment of CAF and TAM subsets with lipid metabolic features in patients with TNBC who exhibited immunotherapy resistance. ABCA8, a key signature gene of lpCAFs, demonstrated specific distribution in lpCAFs through UMAP projection analysis across all cell types and CAFs in two TNBC datasets, confirming its validity as a marker gene (**Supplementary Figures S2E–H**). To examine the relationship between ABCA8^+^ lpCAFs and APOE^+^ LAMs, analysis of TNBC spatial transcriptome data was conducted. The spatial radial distance algorithm from Semla was employed to integrate 43 spatial transcriptome samples and calculate radial distances from each sequencing spot to designated regions. Representative images of immune-stromal junction regions c4 are shown in **Figure 5A**. Analysis of radial distances between cell subset signature genes and regions c4 revealed: T cell marker genes (CD3D, CD3E,CD8A,GZMA,NKG7 and PTPRC) and LAM markers (CD68, APOE) showed relative enrichment within c4, while epithelial markers (EPCAM, KRT18 and KRT8) were distributed outside these regions (**Figures 5B**). The lpCAFs marker ABCA8 consistently demonstrated intra-regional enrichment in c4 regions. Heatmap analysis of differential marker gene distribution across spatial clusters confirmed high co-expression of ABCA8 (lpCAF) with APOE and CD68 (LAM) in c4 (**Figure 5C**), indicating that ABCA8^+^ lpCAFs are concentrated at immune-stromal junctions, suggesting their significance in immune regulation. Representative images demonstrate spatial co-localization of ABCA8^+^ lpCAFs with CD3E and LAM markers (CD68 and APOE) in TNBC tissues (**Figures 5D-F**). Immunofluorescence staining of TNBC samples from patients who failed to achieve pCR after neoadjuvant chemo-immunotherapy confirmed close protein-level co-localization of lpCAFs and LAM, supporting their synergistic roles in TNBC immunotherapy resistance (**Figure 5G**).

### ABCA8^+^ lpCAFs associates with poor prognosis and formation of immunosuppressive microenvironment in TNBC

**Figure 6:**
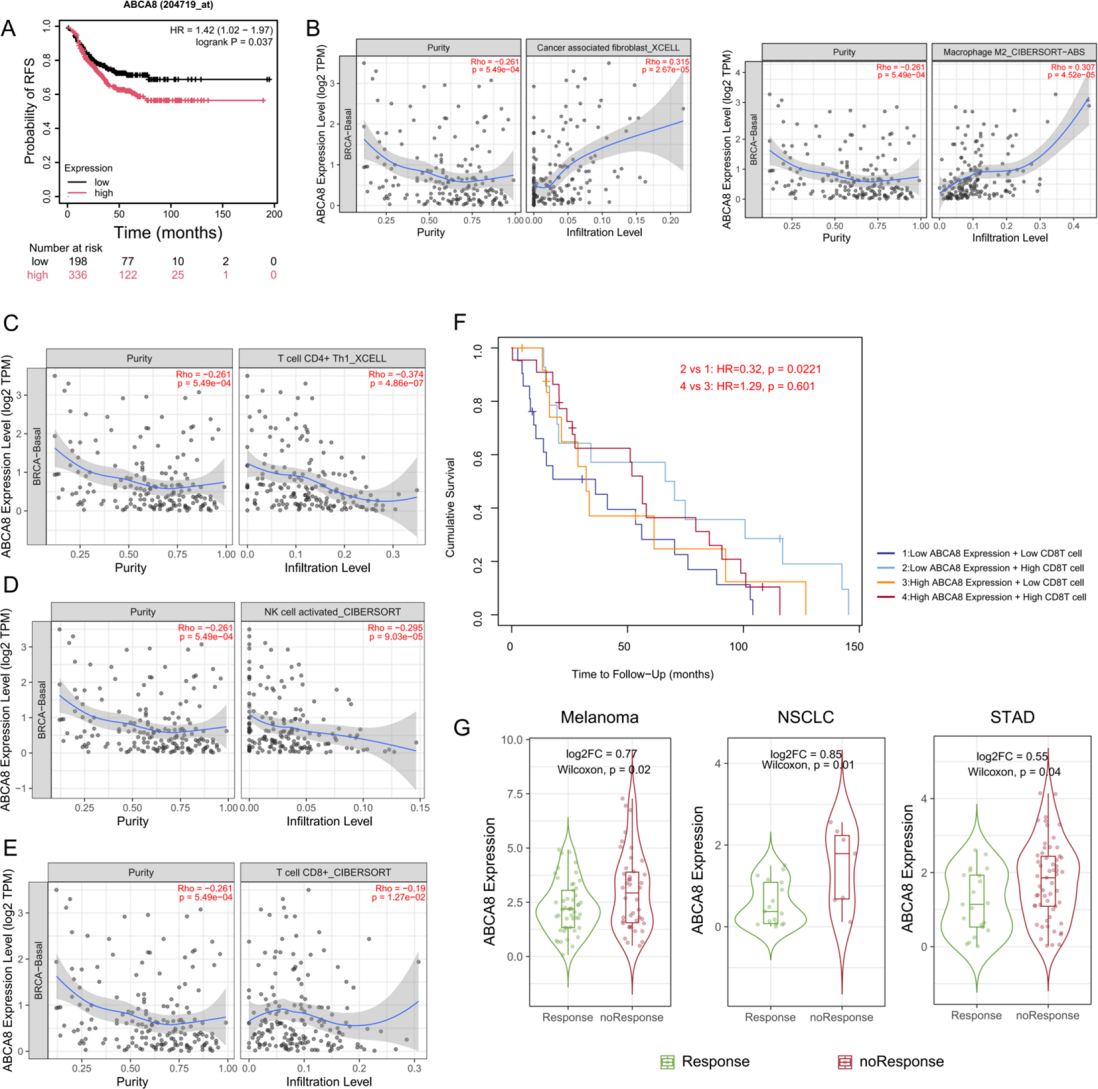
ABCA8 is associated with poor prognosis and immunosuppressive microenvironment in TNBC. **(A)** Kaplan–Meier analysis of Recurrence-Free Survival(RFS)in patients with TNBC stratified by ABCA8 expression. **(B-E)** TIMER3.0 database-based correlation analysis of ABCA8 expression with the abundance of CAFs, Macrophage M2, activated NK cells, CD8+ T cells, and CD4+ Th1 cells, in TNBC breast cancer patients. **(F)** Kaplan-Meier curves displaying OS of 188 patients with TNBC patients stratified by ABCA8 expression and CD8+ T cell infiltration (MCPCOUNTER), with “High” defined as the top 30% for both parameters. The x-axis represents follow-up time in months, and the y-axis indicates cumulative survival rate. **(G)** TIMER3.0 database-based violin plot illustrating the differential expression of ABCA8 in responders and non-responders to immunotherapy in melanoma, NSCLC, and STAD.

The analyses of single-cell transcriptomics and spatial transcriptomics data confirmed that lpCAFs demonstrate significant abundance and spatial correlations with APOE^+^ LAMs displaying immunosuppressive phenotypes. Although lpCAFs, as a key subtype of CAFs, have been linked to poor prognosis and immunosuppressive microenvironments in various tumors, their role in TNBC remains unexplored (34, 35). Survival analysis of patients with TNBC from the KMplot database (ER status - IHC: ER negative; PR status - IHC: PR negative; HER2 status - array: HER2 negative; Dataset: all) revealed that patients with high expression of ABCA8 exhibited poorer Recurrence-Free Survival (RFS)(HR=1.42, logrank P = 0.037), indicating the critical role of lpCAFs in TNBC progression (**Figure 6A**). Bulk RNA transcriptome data analysis showed that high ABCA8 expression positively correlated with increased infiltration of CAFs and M2 macrophages (**Figure 6B**), while negatively correlating with infiltration of NK cells, CD8^+^T cells, and TH1 cells possessing immune-killing functions (**Figures 6C–E**). These results suggest ABCA8 may inhibit immune killing and promote immune evasion in TNBC through regulation of CAFs and M2 macrophages. Survival analysis of patients with TNBC (n = 188) based on ABCA8 expression levels and CD8^+^T cell infiltration demonstrated that high CD8^+^T cell infiltration associated with better overall survival (OS) in patients with low ABCA8 expression, while CD8^+^T cell infiltration showed no significant impact on OS in patients with high ABCA8 expression, suggesting ABCA8 expression may suppress the function of infiltrating CD8^+^T cells (**Figure 6F**). These findings support the hypothesis that ABCA8 functions as a key regulator of the immune microenvironment in TNBC. Analysis of gene expression levels and immunotherapy response correlation using the TIMER3.0 database revealed that high ABCA8 expression is associated with immunotherapy resistance in melanoma, non-small cell lung cancer (NSCLC), and stomach adenocarcinoma (STAD), further indicating ABCA8’s broad role in tumor immune evasion (**Figure 6G**).

### ABCA8^+^ lpCAFs induce lipid metabolic phenotype and M2 polarization of macrophages *In Vitro*​

Bioinformatics analyses have demonstrated that ABCA8^+^ lpCAFs and APOE^+^ LAMs exhibiting lipid metabolic features are co-enriched in TNBC patients with immunotherapy resistance and display close spatial co-localization. To validate their functional association, this study employed a Transwell *in vitro* co-culture model for experimental investigation.

While CAFs exhibit substantial functional heterogeneity, with various CAF subsets identified across different studies, the binary classification based on fibrotic and inflammatory functions has achieved broad consensus. lpCAFs with lipid metabolic features demonstrate significant potential for inflammatory factor secretion, indicating their possible origin from iCAFs with pro-inflammatory functions. Adipose-derived mesenchymal stem cells (AD-SCs) exhibit multi-differentiation potential and self-renewal capacity, functioning as one of the CAF sources *in vivo (*36). Miyazaki et al. demonstrated that tumor cells could induce the differentiation of AD-SCs into iCAFs *in vitro (*23), while Hsu et al. established that pro-adipogenic factors facilitated the transformation of AD-SCs into lpCAFs based on tumor induction of mesenchymal stem cells (37). This study adapted their methodological approaches (detailed in the method section).

**Figure 7:**
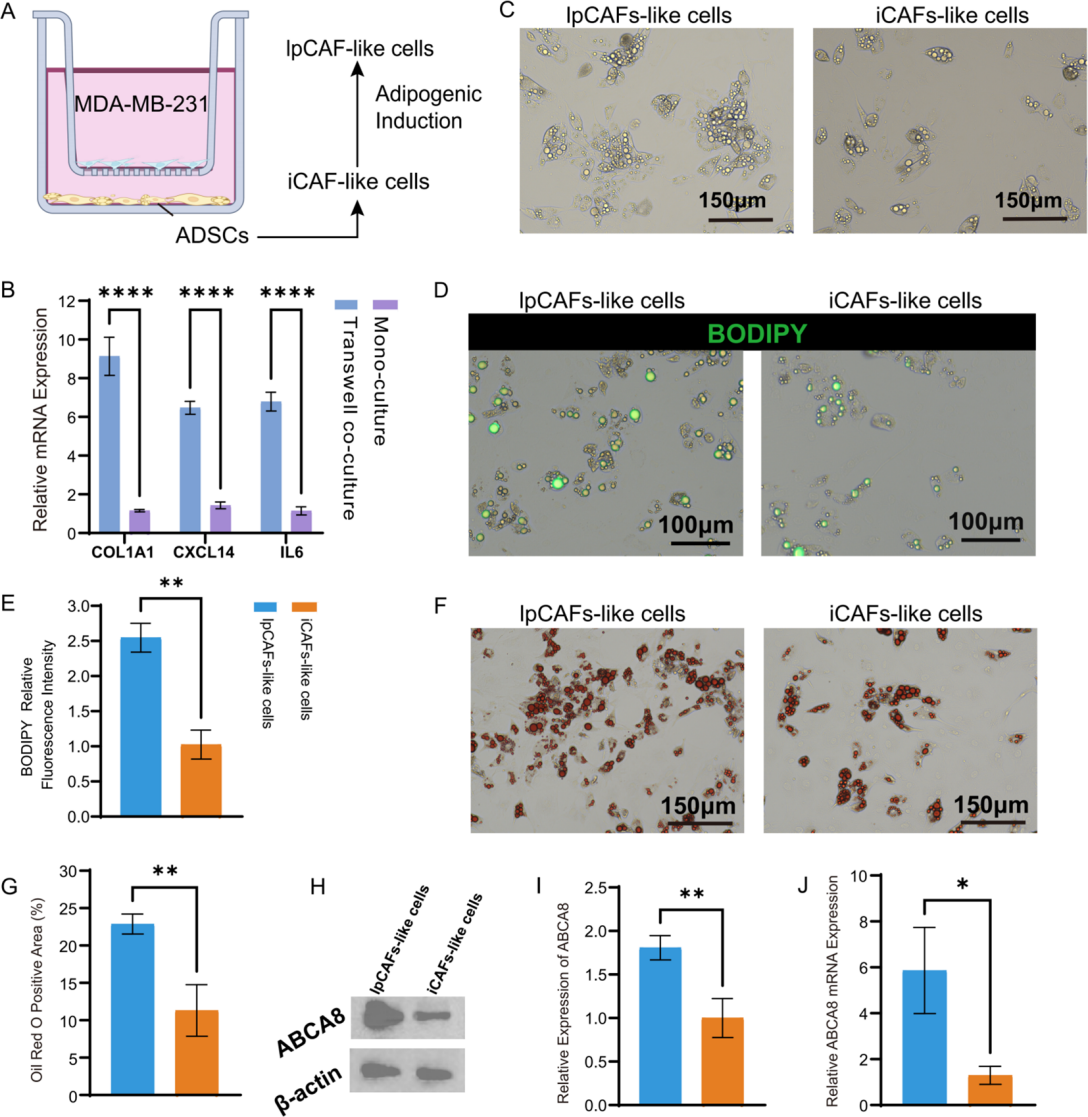
MDA-MB-231 induced ADSCs differentiation into lpCAFs. **(A)** Schematic of MDA-MB-231-induced ADSCs differentiation into iCAFs and lpCAFs. **(B)** Differences in mRNA levels of iCAFs marker genes COL1A1, CXCL14, and IL6 between MDA-MB-231 and ADSCs co-culture group (Transwell co-culture) and ADSCs single culture group (Mono culture). **(C)** Light microscopy observation of cell morphology and lipid droplet synthesis in lpCAFs-like cells and iCAFs-like cells. **(D, E)** BODIPY staining and fluorescence quantification analysis of lpCAFs-like cells and iCAF-like cells (Scale bar=100μm). **(F, G)** Oil red staining and quantitative analysis of chromatin area between lpCAFs-like cells and iCAFs-like cells; **(H, I)** Western blotting detection and quantitative analysis of ABCA8 expression in lpCAFs-like cells and iCAFs-like cells. **(J)** RT-qPCR detection of ABCA8 expression levels in lpCAFs-like cells and iCAFs-like cells. *p<0.05, **p<0.01, ***p<0.001.

In this investigation, AD-SCs isolated from normal breast tissue were co-cultured with the human TNBC cell line MDA-MB-231. The iCAF-like cells were then underwent differentiation into lpCAF-like cells using an adipogenesis induction medium (**Figure 7A**; **Supplementary Figures S2I-N**). Experimental results indicated that compared to untreated ADSCs cultured independently, co-cultured ADSCs showed significantly elevated expression of iCAF marker genes COL1A1, IL6, and CXCL1, confirming their differentiation into iCAF-like cells (**Figure 7B**). Subsequent lipid metabolism analyses revealed marked differences in lipid synthesis capacity between lpCAF-like cells and iCAF-like cells. Light microscopy revealed numerous vacuolar structures surrounding the nuclei in lpCAF-like cells, validating lipid droplet formation (**Figure 7C**). BODIPY staining showed significantly higher normalized fluorescence intensity per cell area in lpCAF-like cells compared to iCAF-like cells (**Figures 7D, E**). Similarly, Oil Red O staining demonstrated a significantly larger percentage of cell area occupied by lipid droplets in lpCAF-like cells (**Figures 7F, G**). Western blotting and RT-qPCR confirmed significantly increased ABCA8 expression in lpCAF-like cells relative to iCAF-like cells (**Figures 7H–J**). These results confirmed that ADSCs co-cultured with MDA-MB-231 could differentiate into lpCAFs with high ABCA8 expression and lipid metabolism characteristics. Compared to iCAFs, lpCAFs demonstrated higher ABCA8 expression and enhanced lipid generation capacity.

**Figure 8:**
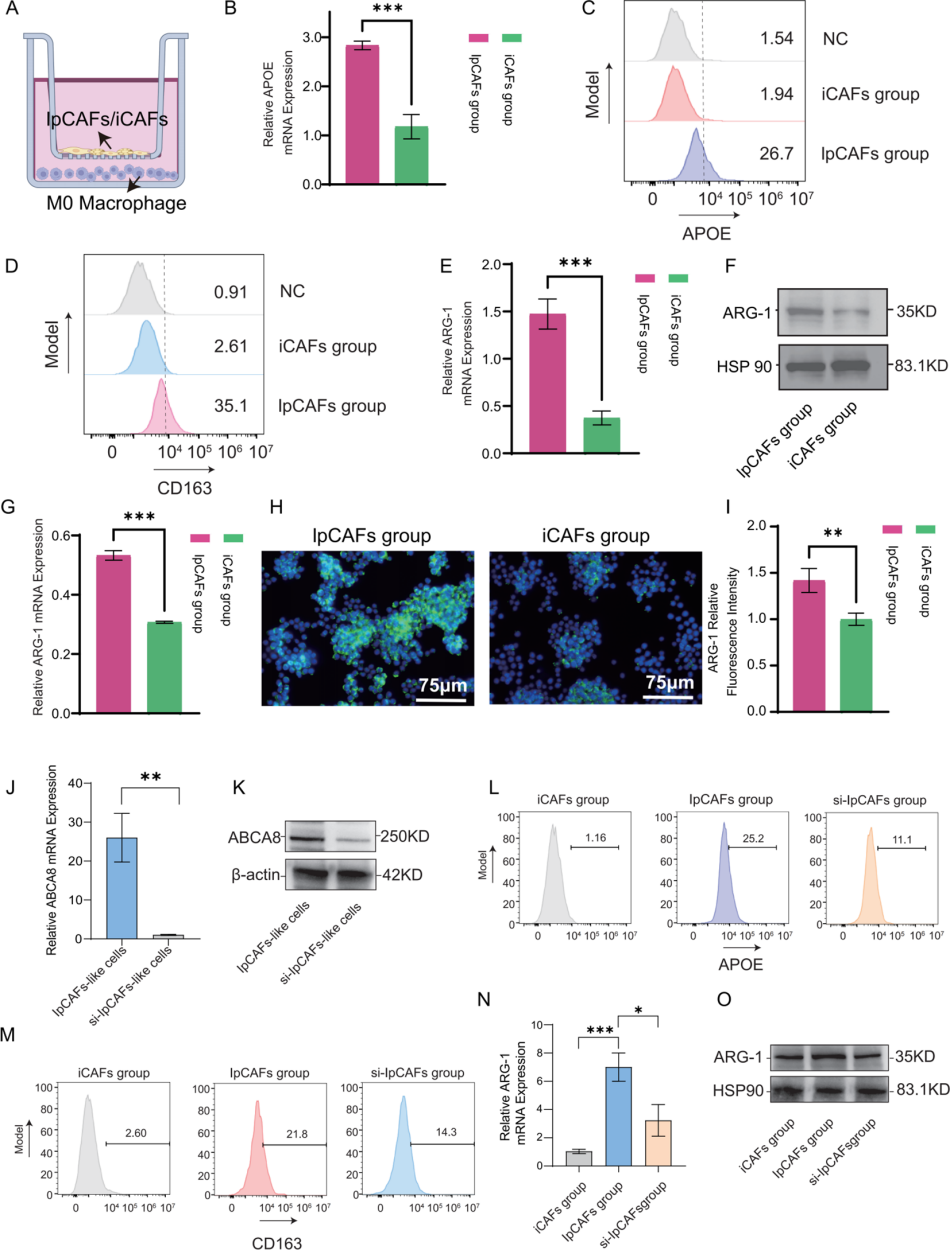
MDA-MB-231 induced ADSCs differentiation into lpCAFs to promote macrophage M2 polarization. **(A)** Schematic of co-culture between lpCAFs-like cells or iCAFs-like cells and M0 macrophages. **(B)** RT-qPCR detection of APOE mRNA levels in macrophages following co-culture with lpCAFs-like cells or iCAFs-like cells. **(C)** Flow cytometry analysis showing APOE expression levels in macrophages after co-culture with iCAFs-like cells or lpCAFs-like cells. **(D)** Flow cytometry analysis showing CD163 expression levels in macrophages after co-culture with iCAFs-like cells or lpCAFs-like cells. **(E)** RT-qPCR detection of ARG-1 mRNA levels in macrophages following co-culture with lpCAFs-like cells or iCAFs-like cells. **(F, G)** Western blotting detection and quantitative analysis of ARG-1 expression levels in macrophages following co-culture with lpCAFs-like cells or iCAFs-like cells. **(H)** Immunofluorescence staining of ARG-1 in M0 macrophages after co-culture with lpCAFs or iCAFs (Scale bar=75μm). **(I)** Quantitative analysis of ARG-1 immunofluorescence staining following co-culture of lpCAFs-like cells or iCAFs-like cells with M0 macrophages. **(J)** RT-qPCR results showing the mRNA levels of ABCA8 in lpCAFs cells and lpCAFs treated with ABCA8-siRNA. **(K)** Western blotting results showing the protein expression levels of ABCA8 in lpCAFs and lpCAFs treated with ABCA8-siRNA. **(L)** Flow cytometry results showing the APOE expression levels in macrophages after co-culture with iCAFs, lpCAFs or lpCAFs treated with ABCA8-siRNA. **(M)** Flow cytometry results showing the CD163 expression levels in macrophages after co-culture with iCAFs, lpCAFs or lpCAFs treated with ABCA8-siRNA. **(N)** RT-qPCR results showing the mRNA levels of ARG-1 in macrophages after co-culture with iCAFs, lpCAFs, or lpCAFs treated with ABCA8-siRNA. **(O)** Western blotting results showing the protein levels of ARG-1 in macrophages after co-culture with iCAFs, lpCAFs, or lpCAFs treated with ABCA8-siRNA. *p<0.05, **p<0.01, ***p<0.001.

To validate the M2-polarizing effect of lpCAFs on macrophages, PMA was used to induce THP-1 monocytes into macrophage-like cells. These were then co-cultured with induced lpCAFs or iCAFs using a Transwell system (**Figure 8A**). We examined the expression of APOE—a lipid metabolism marker in macrophages identified in prior single-cell omics analyses. RT-qPCR and flow cytometry results indicated that lpCAFs significantly upregulated both transcriptional and protein levels of APOE in M0 macrophages (**Figures 8B, C**). Furthermore, flow cytometry detection of another lipid metabolism marker, compared to the isotype control antibody, there was no significant difference in CD163 expression in the iCAF group, whereas CD163 expression in the ipCAF group was significantly upregulated (**Figure 8D**). To assess the extent of M2 polarization, we further detected the expression of ARG-1, a classic M2 macrophage marker. RT-qPCR (**Figure 8E**), Western blot (**Figures 8F, G**), and immunofluorescence (**Figures 8H, I**) results consistently demonstrated significantly elevated ARG-1 expression in macrophages co-cultured with lpCAFs, indicating that lpCAFs promote M2 polarization.

To further evaluate the role of ABCA8 in lpCAF-induced M2 polarization, we performed siRNA-mediated knockdown of ABCA8. RT-qPCR (**Figure 8J**) and Western blot (**Figure 8K**) confirmed efficient reduction of ABCA8 expression in lpCAFs. We then co-cultured M0 macrophages with iCAFs, lpCAFs, or ABCA8-silenced lpCAFs (si-lpCAFs) to assess their polarizing capacity. Flow cytometry revealed that, compared to the lpCAF group, macrophages co-cultured with si-lpCAFs showed significantly reduced expression of lipid metabolism markers APOE and CD163 (**Figures 8L, M**). Additionally, qPCR (**Figure 8N**) and Western blot (**Figure 8O**) indicated markedly decreased expression of the M2 marker ARG-1. These findings suggest that ABCA8 knockdown effectively attenuates the reprogramming of macrophage lipid metabolism and M2 polarization induced by lpCAFs.

## Discussion​

Immune checkpoint inhibitors (ICBs) have fundamentally transformed cancer therapy through their ability to induce durable immune responses, demonstrating remarkable efficacy in various solid tumors, including breast cancer, lung cancer, and melanoma (38). The application of ICBs to TNBC has emerged as a major area of research in recent years. The U.S. Food and Drug Administration (FDA) has approved pembrolizumab in combination with chemotherapy for neoadjuvant treatment of high-risk early-stage TNBC and metastatic TNBC. However, the results of the landmark KEYNOTE-522 clinical trial revealed that more than 30% of patients with early-stage TNBC did not achieve pCR after neoadjuvant treatment (39). The tumor response to immunotherapy depends heavily on complex synergistic interactions among components within the TME. Therefore, a comprehensive understanding of the cellular interaction network within the TME is essential for improving patient prognosis. This study integrated single-cell omics and spatial transcriptomics analyses of TNBC to classify tumor niches based on the transcriptomic characteristics of each spot. The analysis revealed that T cells, which are targets of immunotherapy, concentrate in immune niches and immune-stromal junction niches. Cell communication analysis based on spatial transcriptomics demonstrates that the immune interstitial junction region exhibits complex and active cellular interactions. These findings indicate that the spatial organization of TNBC niches, particularly the immune-stromal junction enriched with T/NK cells, myeloid cells, and fibroblasts, functions as a regulatory hub for intercellular communication.

Macrophages, which represent the most abundant and active cells in the TME, have been extensively documented for their immunosuppressive role. The M1/M2 polarization model originated from the response of macrophages to different stimuli. It has been instrumental in distinguishing between the pro-inflammatory (M1) and anti-inflammatory (M2) phenotypes of macrophages and is widely used in tumor microenvironment (TME) research to characterize the function of TAMs (40). While this model has been validated and gained consensus, it represents an oversimplification of macrophage heterogeneity. Early studies relied on *in vitro* stimulation to induce polarization; however, *in vivo* macrophages often co-express genes associated with both M1 and M2 phenotypes, indicating their states are mixed and plastic. Therefore, the M1/M2 framework should be considered a useful starting point rather than a definitive classification system. The application of high-throughput technologies like scRNA-seq has allowed for the resolution of TAM heterogeneity at the transcriptome level. These studies reveal that macrophages in the TME form multiple functional subsets with distinct transcriptional regulatory networks, exhibiting either tumor-promoting or tumor-suppressing functions. Another key advancement in macrophage biology is the recognition of the heterogeneity in their origin (ontogeny), which directly influences their functional properties. TAMs primarily derive from two lineages: tissue-resident macrophages (TRMs), which originate from embryonic precursors, and monocyte-derived macrophages (MDMs), which are recruited to the tumor site via chemotactic signals such as CCL2 and CSF-1. This difference in origin critically shapes their functional specialization within the TME (41). In conclusion, macrophages exhibit remarkable heterogeneity, which must be carefully considered and discussed within specific pathological and microenvironmental contexts.

Analysis of TNBC single-cell datasets with clear prognostic information on immunotherapy revealed that a subset of TAMs (LAMs), characterized by both lipid metabolic and immune-negative regulatory features, showed significant enrichment in patients with immunotherapy resistance. The immunosuppressive phenotype of macrophages is strongly correlated with their metabolic reprogramming, particularly in lipid metabolism. This metabolic shift enables TAMs to modulate immune responses, creating an environment conducive to tumor progression and resistance to immunotherapy. Based on spatial transcriptomics data, this study observed significant spatial co-localization between macrophages and stromal cells, represented by CAFs, in the immune-stromal junction area. Furthermore, ligand-receptor interaction analysis in single-cell data revealed that among all signals received by macrophages, the signal intensity from CAFs increased significantly in the immunotherapy-resistant group. This finding strongly indicates the critical role of CAF-macrophage crosstalk in regulating TNBC immunotherapy response. These discoveries elucidate a novel cellular interaction axis contributing to ICB resistance, suggesting potential biomarkers and therapeutic targets for improving TNBC outcomes.

In-depth clustering analysis of CAFs identified a subset, lpCAFs, which was also specifically enriched in the resistant group. This subgroup has lipid metabolism characteristics and high expression of ABCA transporters. Further results based on large-scale single-cell sequencing indicate that ABCA8 is its specific marker. Previous literature has reported that ABCA8 is a key lipid transporter that promotes tumor cell proliferation and drug resistance by mediating lipid efflux in pancreatic cancer (42). This study identified the ABCA8^+^ lpCAFs subgroup in TNBC for the first time, and proved that ABCA8^+^ lpCAFs are associated with poor long-term prognosis of TNBC and immunotherapy resistance. Based on single-cell subpopulation clustering results and pseudo-temporal differentiation trajectories, lpCAFs exhibited characteristics and differentiation pathways similar to iCAFs, while exhibiting significant differences from myCAF. We speculate that lpCAFs may have a similar origin to iCAFs; our study referred to the previous methods of inducing ADSC differentiation to induce lpCAFs (23, 36). Co-culture of ADSCs with TNBC tumor cells induced their differentiation into iCAF-like cells, which were then transformed into lpCAFs-like cells with higher lipid synthesis capacity and high ABCA8 expression under adipogenesis induction.

The spatial proximity of ABCA8^+^ lpCAFs and APOE^+^ LAMs at the immune-stromal junction, confirmed through spatial transcriptomics and immunofluorescence, indicates a localized regulatory hub where lipid transport by lpCAFs facilitates metabolic reprogramming of macrophages. This aligns with the established role of lipid metabolism in modulating immune cell function, as lipids provide substrates that promote M2 polarization, thereby reducing pro-inflammatory activity. Previous literature has reported that in the tumor microenvironment of breast cancer, the interaction between cancer-associated fibroblasts (CAFs) and tumor-associated macrophages (TAMs)drives immune suppression and therapeutic resistance through a complex paracrine signaling network. CAFs secrete various factors (e.g., IL-10, CCL2, SDF-1/CXCL12) to recruit monocytes, which are then polarized into immunosuppressive TAMs via pathways such as Jagged1/Notch. In turn, immunosuppressive TAMs activate CAFs by producing mediators such as IL-6, TGF-β, and prostaglandin E2 (PGE2), forming a positive feedback loop that enhances epithelial-mesenchymal transition (EMT) and extracellular matrix remodeling, thereby promoting tumor progression and reducing the efficacy of immune checkpoint inhibitors (43).

Our research demonstrated that lpCAF-like cells effectively induce lipid metabolism activation and M2 phenotype polarization in THP-1-derived MO macrophages, highlighting the essential role of lpCAFs in orchestrating immune microenvironmental balance. Our study provides a novel perspective that ABCA8, which is specifically highly expressed in CAFs, becomes a new key regulator of TAM function—by mediating lipid efflux to trigger lipid metabolism reprogramming within macrophages, promoting lipid accumulation and inducing immunosuppressive phenotypic polarization. This metabolic remodeling synergizes with factors secreted by CAFs further amplifying the immunosuppressive phenotypes of macrophages. This mechanism reveals a new paradigm of CAFs reprogramming TAMs through lipid metabolism, offering a novel target for overcoming resistance to immune checkpoint inhibitors.

Our study has several limitations. For instance, conclusions derived from *in vitro*models need to be further validated in complex *in vivo*environments, and the immunosuppressive phenotype of macrophages following lipid metabolism reprogramming may require additional indicators for comprehensive characterization. Future studies should elucidate the specific molecular pathways through which ABCA8 regulates lipid metabolism, such as its effects on the exocytosis or intracellular transport of specific lipid molecules. Our validation of ABCA8 was based on cell models, which has certain limitations. To further validate its role in reprogramming macrophage lipid metabolism, more robust platforms such as organoid models simulating the tumor microenvironment should be utilized. On this basis, the development of selective small-molecule inhibitors or modulators of ABCA8 will provide new and valuable options for the synergistic use of immunotherapies.

## Data Availability

The original contributions presented in the study are included in the article/**Supplementary Material**. Further inquiries can be directed to the corresponding author/s.

## Supplementary material

Supplementary Figure 1 **(A)** Bar chart showing the GO enrichment results of c4 region differential genes. **(B)** Box plot showing the proportion that myeloid subsets accounts for myeloid cells in the T cell amplification group (E) and the non-amplification group (NE) before and after treatment, the Wilcox test, * represents p <0.05. **(C)** Bubble plot showing feature gene distribution across clusters. Bubble size represents the percentage of cells expressing marker genes within each cluster, and the color gradient indicates normalized mean expression levels. **(D)** Bar chart showing the GO enrichment results of LAM differential genes.

Supplementary Figure 2 **(A)** The chord diagram shows the difference in signal intensity between NE group and E group targeting myeloid cells. The thickness of the lines represents the difference in intensity, with red representing enhanced in NE group and blue representing weakened in NE group. **(B)** The dendrogram of WGCNA shows the clustering pattern of genes. Different colors represent different expression patterns, and gray represents genes that cannot be clustered into modules. **(C)** The violin plot shows the distribution differences of top30 M2 module genes scores among different CAFs subtypes. **(D)** Bubble plot shows the distribution of the TOP30 genes of the M2 module calculated by hdWGCNA in various CAFs subtypes. **(E, F)** The density plot shows the distribution of ABCA8 gene in all the cells of the tumor microenvironment **(E)** and in CAFs **(F)**, using datasets from VIB-KU Leuven Cancer Biology Center. **(G, H)** The density plot shows the distribution of ABCA8 gene in all the cells of the tumor microenvironment **(G)** and in CAFs **(H)**, using datasets from GSE246613. **(I)** Light microscopy observation of cell morphology of ADSCs, which exhibited typical spindle-shaped morphology. **(J-N)** Flow cytometry results showed that the extracted cells expressed CD90 (98.3%, **J**), CD44 (97.6%, **K**), and CD29 (98.3%, **L**), but did not express CD45 (1.8%, **M**) and CD34 (1. 5%, **N**), consistent with the characteristics of ADSCs.
