# Supplementary figures and images for: ABCA8-positive lipid-metabolic CAFs mediate immunotherapy resistance in TNBC
Source: Front Oncol. 2026 Jan 27;15:1729275. doi: 10.3389/fonc.2025.1729275 (PMC12887699; doi:10.3389/fonc.2025.1729275)

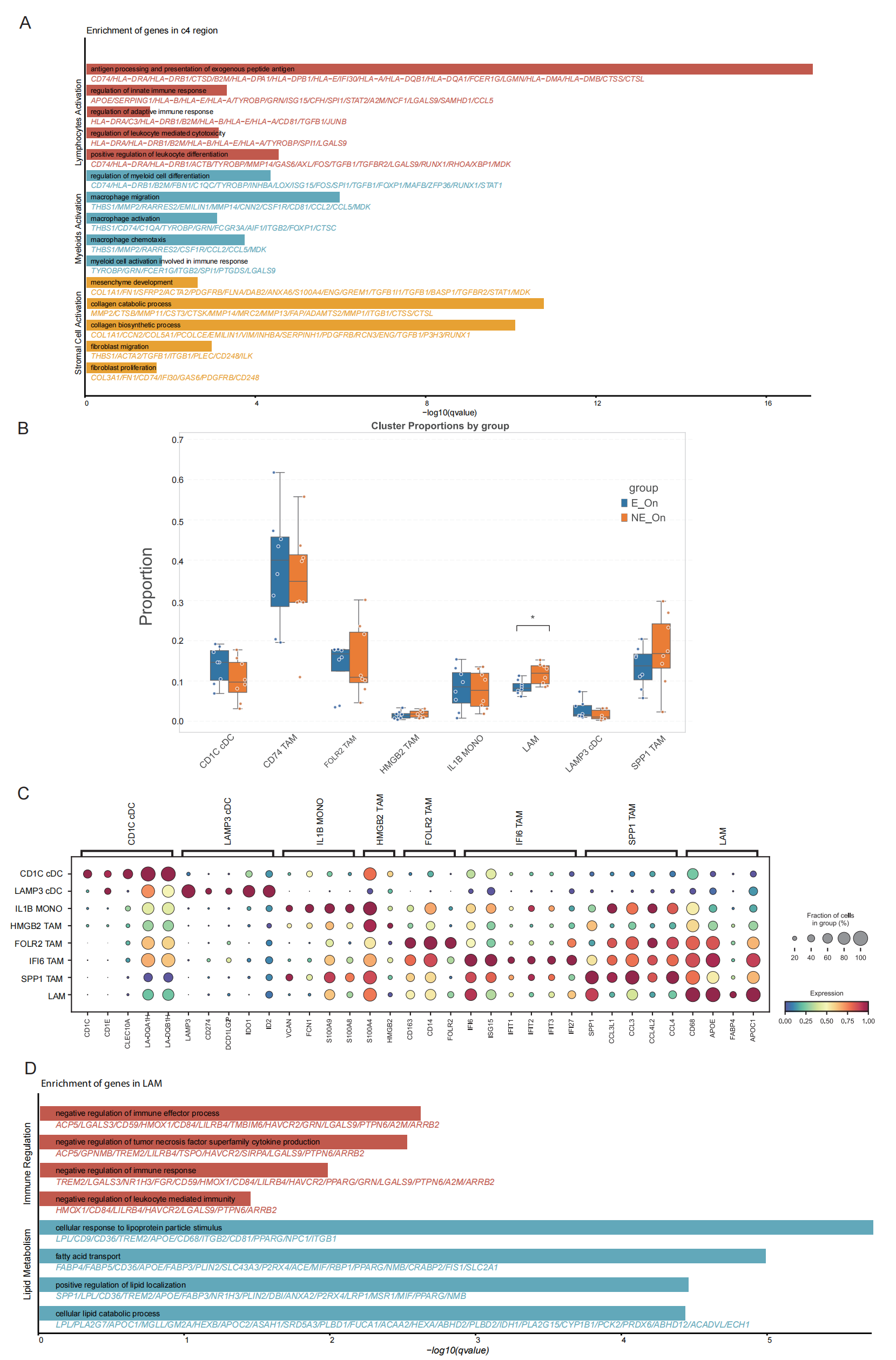

Supplement: Supplementary Figure 1 — (A) Bar chart showing the GO enrichment results of c4 region differential genes. (B) Box plot showing the proportion that myeloid subsets accounts for myeloid cells in the T cell amplification group (E) and the non-amplification group (NE) before and after treatment, the Wilcox test, * represents p <0.05. (C) Bubble plot showing feature gene distribution across clusters. Bubble size represents the percentage of cells expressing marker genes within each cluster, and the color gradient indicates normalized mean expression levels. (D) Bar chart showing the GO enrichment results of LAM differential genes. [file Image1.tif]

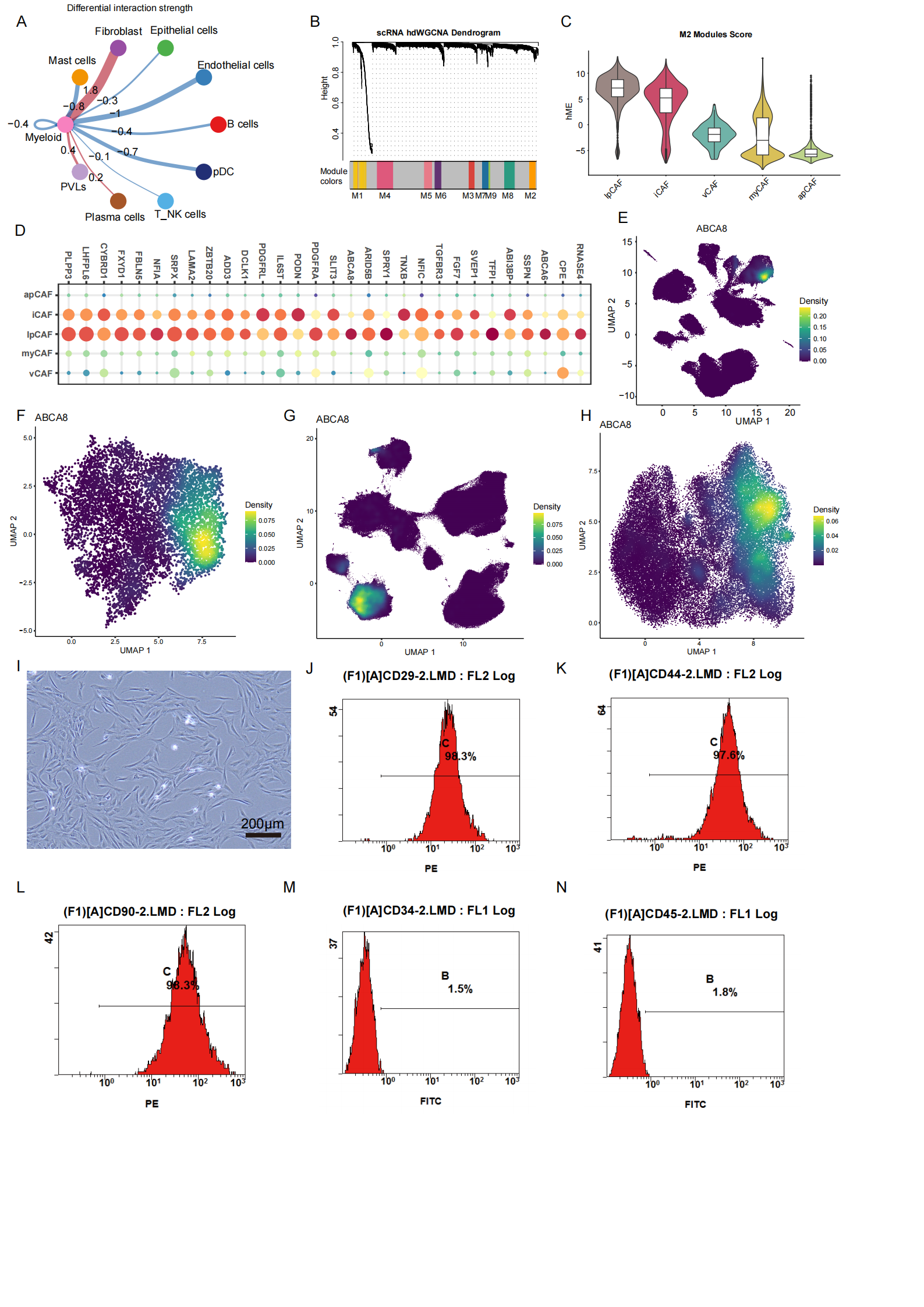

Supplement: Supplementary Figure 2 — (A) The chord diagram shows the difference in signal intensity between NE group and E group targeting myeloid cells. The thickness of the lines represents the difference in intensity, with red representing enhanced in NE group and blue representing weakened in NE group. (B) The dendrogram of WGCNA shows the clustering pattern of genes. Different colors represent different expression patterns, and gray represents genes that cannot be clustered into modules. (C) The violin plot shows the distribution differences of top30 M2 module genes scores among different CAFs subtypes. (D) Bubble plot shows the distribution of the TOP30 genes of the M2 module calculated by hdWGCNA in various CAFs subtypes. (E, F) The density plot shows the distribution of ABCA8 gene in all the cells of the tumor microenvironment (E) and in CAFs (F), using datasets from VIB-KU Leuven Cancer Biology Center. (G, H) The density plot shows the distribution of ABCA8 gene in all the cells of the tumor microenvironment (G) and in CAFs (H), using datasets from GSE246613. (I) Light microscopy observation of cell morphology of ADSCs, which exhibited typical spindle-shaped morphology. (J-N) Flow cytometry results showed that the extracted cells expressed CD90 (98.3%, J), CD44 (97.6%, K), and CD29 (98.3%, L), but did not express CD45 (1.8%, M) and CD34 (1. 5%, N), consistent with the characteristics of ADSCs. [file Image2.tif]
